# Radiodermatitis and Fibrosis in the Context of Breast Radiation Therapy: A Critical Review

**DOI:** 10.3390/cancers13235928

**Published:** 2021-11-25

**Authors:** Sofiane Allali, Youlia Kirova

**Affiliations:** Radiation Therapy Department, Institut Curie, CEDEX 05, 75248 Paris, France

**Keywords:** radiation therapy, breast cancer, skin toxicities, fibrosis, radiodermatitis, radiotherapy, IMRT, 3D

## Abstract

**Simple Summary:**

Skin toxicity is the main complication during irradiation in the management of early-stage breast cancer. In some cases, it may cause treatment to stop. These toxicities may be acute (mainly radiodermatitis) and/or late (mainly fibrosis). Their understandings, their mechanisms of occurrence, as well as their management is indispensable in order to improve the management of these patients. Through this study we propose to provide a clear picture of these toxicities in relation to the modalities of radiotherapy, advances in their quantification, and management to help practitioners improve their knowledge and clinical practices on this topic.

**Abstract:**

Background: Radiation therapy has been progressively improved in order to maintain a satisfactory tumour response, while reducing toxicity. We will review the incidence of radiodermatitis and fibrosis according to the various radiation and fractionation techniques. We will then focus on the various methods used to manage, prevent, and quantify this toxicity. Method: More than 1753 articles were identified using the various search terms. We selected 53 articles to answer the questions addressed in this study according to criteria set in advance. Result: The literature reports lower acute toxicity with IMRT compared to 3DCRT, but no significant differences in terms of late toxicities. Partial breast irradiation appears to be less effective in terms of local control with a higher rate of late toxicity. Intra operative radiation therapy appears to provide good results in terms of both local control and late toxicity. The hypofractionation has equivalent efficacy and safety to the normofractionated regimen, but with lower rates of radiodermatitis and fibrosis. The adddition of a boost, particularly a sequential boost, increases the risk of fibrosis and radiodermatitis during treatment. Conclusion: The development of IMRT has significantly reduced acute toxicity and has improved tolerability during treatment. Modified fractionation has reduced treatment time, as well as adverse effects.

## 1. Introduction

Considerable progress has been made in the field of radiation therapy over recent years, both in terms of medical physics, by replacing the use of cobalt by other particles or radiation such as photons, protons, and other forms of particle therapy, and in terms of radiation techniques, with two-dimensional (2D) and then three-dimensional (3D) conformal radiation therapy, followed by intensity-modulated radiation therapy (IMRT) [1] and the use of additional techniques such as lateral decubitus position [2] or deep inspiration breath hold [3,4]. The aim of radiation therapy is therefore no longer simply the control and eradication of tumours, but also preservation of healthy organs and reduction of toxicities and cosmetic sequelae. Radiation therapy has therefore been progressively improved in order to maintain a satisfactory tumour response, while reducing toxicity and preserving the appearance of the breast.

The main skin toxicities of radiation therapy of eBC are classified into two categories: acute toxicities (<6 months), mainly consisting of radiodermatitis, and late toxicities (>6 months) mainly consisting of fibrosis. These toxicities are evaluated clinically and graded according to international classifications CTCAE v4 or v5 [5], RTOG [6], etc.

The current challenge of radiation therapy is therefore to maintain good tumour response, while minimizing side effects such as radiodermatitis and fibrosis.

In this study, we will review the incidence of radiodermatitis and fibrosis in breast cancer according to the various radiation and fractionation techniques. We will then focus on the various methods used to manage, prevent, and quantify this toxicity.

## 2. Methods

This study was carried out by searching for articles in PubMed, Medline, and Google Scholar using the MESH terms: “fibrosis”; “breast cancer”, “radiation therapy” or “radiotherapy”, “radiodermatitis”, “skin toxicity AND radiotherapy”, “skin toxicity AND radiotherapy AND breast cancer”, “fibrosis AND radiotherapy”, “radiodermatitis AND radiotherapy”, “fibrosis AND radiotherapy AND breast cancer”, “radiodermatitis AND radiotherapy AND breast cancer”.

The population of interest is defined as patients treated for early breast cancer and receiving radiation therapy. The aim of the study is to present a state-of-the-art report on the occurrence of acute and late skin toxicities in relation to the different modalities of radiotherapy, and to highlight evaluation practices, analysis, management, and prevention of these toxicities through a literature review.

**Figure 1:**
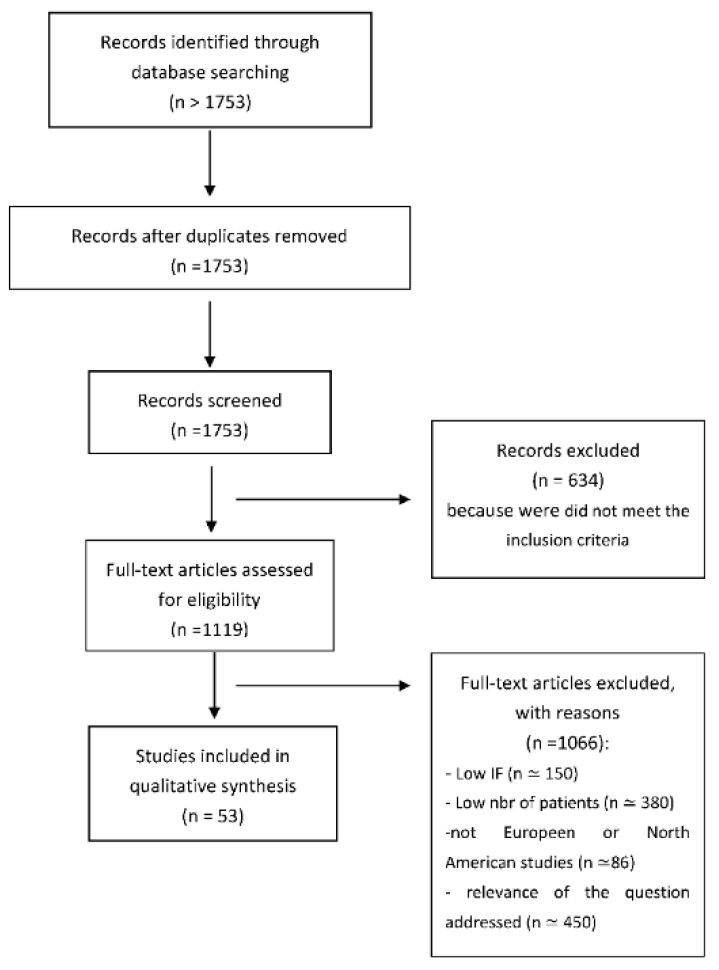
Flow Chart.

No limit concerning the date of publication of the articles was defined, and articles that did not exclusively concern breast cancer, articles using radiation that is no longer used routinely (Cobalt, brachytherapy, prone position, proton therapy, carbon therapy), articles evaluating toxicities without using international CTCAE [7] or RTOG [6] criteria in the case report, and studies that did not assess fibrosis and/or radiodermatitis, were excluded. More than 1753 articles were identified using the various search terms, more than 634 of which did not meet the inclusion criteria. We therefore selected 53 articles to answer the questions addressed in this study according to the impact factor of the journal where they were published (IF), and the relevance of the question addressed in each study, according to the number of patients studied and focusing on North American and European studies (Figure 1).

## 3. Results

### 3.1. Pathophysiology

#### 3.1.1. Radiodermatitis

The interaction between ionizing radiation and tissues results in the formation of free radicals, either via a direct action on molecules or via products of water radiolysis. These free radicals induce oxidative stress that can cause chemical, structural, and functional changes in immediate contact with organic molecules. As skin is a hierarchical tissue, distinct cellular compartments are responsible for cellular renewal (stem cells) and function (mature cells). A variable proportion of stem cells, depending on the dose, die following irradiation. However, loss of these cells does not initially induce any detectable lesions, which are masked by differentiated cells. When differentiated cells die from senescence, the residual number of stem cells is insufficient to compensate for physiological turnover of senescent cells. Tissue lesions then become clinically detectable. The interval between radiation therapy and expression of radiodermatitis is therefore dependent on the lifespan of mature cells. Radiodermatitis constitutes an important factor affecting treatment tolerability, as it determines the normal course of radiation therapy, and more severe radiodermatitis may require discontinuation of treatment [8,9].

#### 3.1.2. Fibrosis

Histological features of radiodermatitis range from inflammation to dermal sclerosis. Radiation-induced lesions of both endothelium and connective tissue, amplified by the action of growth factors, are responsible for remodelling and persistence of fibrous tissue. Several phases of fibrosis have been described [10,11].

The prefibrotic phase is characterized by chronic inflammation. Endothelial cells play an important role in the constitution of the prefibrotic phase. Inflammation is characterized by increased vascular permeability, resulting in extravasation of serum proteins and the formation of oedema. Destruction of endothelial cells and associated vascular thrombosis lead to necrosis of microvessels, resulting in local ischaemia, triggering an inflammatory reaction.

At the fibrotic phase, fibrous tissue is composed of inflammatory cells and fibroblasts. Endothelial cells remain present during these secondary neovascularization phenomena related to extension of fibrosis. Zones of active fibrosis are observed, characterized by the presence of extracellular matrix, myofibroblasts, and neovessels. Fibrous tissue becomes increasingly dense as a result of successive remodelling of the extracellular matrix during late phases of reactivated acute inflammation [11].

### 3.2. Radiation Techniques (3DCRT, IMRT, IORT, PBI/APBI)

With progress in computers and robotics and a better knowledge of medical physics, radiation therapy has evolved by proposing new radiation techniques allowing optimization of radiation therapy.

In the vast majority of cases, breast radiation therapy is delivered by means of 3D techniques, especially in the case of single breast radiation therapy [12]. In expert centres, eligible patients can be treated in the lateral decubitus position [2] to limit irradiation of healthy organs such as the lung and the heart. The development of IMRT [1] has allowed improvement of treatment tolerability by significantly limiting the dose delivered to organs at risk (OAR). At the present time, IMRT is only used for breast radiation therapy in patients with specific anatomy (pectus valgus) or for bilateral lymph node or breast radiation therapy [12].

Many studies have compared the toxicity of 3D conformal radiation therapy vs. IMRT [13,14,15,16].

The multicentre, double-blind, randomized controlled trial conducted by Pignol et al. [13] evaluated about 330 patients with breast cancer, treated by either IMRT or conventional radiation therapy with a protocol of 50 Gy in 25 fractions ± a 16 Gy boost. This study demonstrated a significant reduction of the radiodermatitis rate in the IMRT arm. In particular, 31.2% of patients treated by IMRT experienced acute dermatitis compared to 47.8% of patients in the standard treatment arm. However, no statistically significant difference in terms of quality of life was observed in this study. These results were confirmed by other authors, including Krug et al. [14], who demonstrated a higher rate of radiodermatitis in the 3DCRT arm compared to the IMRT arm, but with no significant differences in terms of pain and dysaesthesia between the two arms.

The phase III, multicenter, prospective randomized trial conducted by Horner-Rieber et al. [15] in 502 patients evaluated IMRT breast radiation therapy with integrated boost (50.4 Gy/64.4 Gy) vs. 3DCRT with sequential boost (50.4 Gy/66.4 Gy). With a follow-up of 2 years, cosmetic evaluation using the Harvard and LENT SOMA criteria [17] did not demonstrate any significant difference in terms of atrophy/retraction, telangiectasia, fibrosis, lymphoedema, or breast oedema between the two arms.

These clinical results are also confirmed by dosimetric data. The literature therefore reports lower rates of acute toxicity (radiodermatitis) with IMRT compared to 3DCRT, but no significant differences in terms of late toxicities, especially fibrosis (Table 1).

Some authors have also evaluated various IMRT techniques, particularly Tomotherapy [18,19,20]. Lee et al. [19] evaluated 216 breast cancer patients treated by either IMRT or Tomotherapy with an integrated boost regimen (50.4 Gy/60.2 Gy) with a median follow-up of 5 years. Fewer patients experienced radiodermatitis (grade 2 to 3) in the Tomotherapy arm [2.4% vs. 16% in the IMRT arm (*p* = 0.021)]. No statistically significant difference in terms of long-term toxicities, including fibrosis, was observed between the IMRT and Tomotherapy arms (*p* = 0.57).

Other radiation techniques have also been analysed, such as partial breast radiation [21,22,23,24] or intraoperative radiation therapy [25,26,27,28], which are designed to limit irradiation to the surgical site.

**Table 1:** Acute and late toxicities depending on the irradiation modality.

Authors	No. of Patients	Cancer Location	Radiation Dose (Gy)	No. of Fractions	Boost Dose (Gy)	Irradiation Modality	Radiodermatitis ≥ Grade 2	Fibrosis ≥ Grade 2	Cosmetic Satisfaction ≥ Good
Pignol et al. [13]	330	breast cancer	50	25	16	3D vs. IMRT	47.8% vs. 31.2% (*p* = 0.02)	NA	NA
Krug et al. [14]	446	breast cancer	50.4	28	16	3D vs. IMRT	29.1% vs. 20.1% (*p* = 0.02)	NA	NA
Hörner-Rieber et al. [15]	502	breast cancer	50.4	28	14–16	3D vs. IMRT	NA	10.4% vs. 11.5% (*p* = 0.27)	77.5% vs. 77.3% (*p* = 0.33)
Askoxylakis et al. [16]	502	breast cancer	50.4	28	14–16	3D vs. IMRT	47.8% vs. 31.2% (*p* < 0.05)	NA	NA
Lee et al. [19]	216	breast cancer	50.4	28	9.8	IMRT vs. Tomo	16% vs. 2.4% (*p* = 0.02)	1.7% vs. 2.4% (*p* = 0.57)	NA
Joseph et al. [20]	177	breast cancer	50	25	NA	IMRT vs. Tomo	33% vs. 11% (*p* < 0.001)	75% vs. 67% (*p* = 0.13)	NA
McCormick et al. [23]	2232	breast cancer	40–56 vs. 20	NA	NA	WBRT vs. PBI	NA	NA	NA
Falco et al. [25]	150	breast cancer	46–50 vs. 20	23–25 vs. 1	NA	IORT vs. IORT + WBRT	NA	1.4% vs. 23% (*p* < 0.001)	NA
Key et al. [26]	41	breast cancer	46–50.4 vs. 20	23–28 vs. 1	NA	IORT vs. IORT + WBRT	NA	2.4% vs. 43.3% (*p* < 0.001)	67.3% vs. NA
Sperk et al. [27]	305	breast cancer	46–50 vs. 20	23–25 vs. 1	NA	IORT vs. IORT + WBRT	NA	5.9% vs. 37.5% (*p* < 0.001)	NA
Kraus et al. [28]	73	breast cancer	46 + 20	23 + 1	NA	IORT vs. IORT + WBRT	NA	25%	>90%

As demonstrated by Hickey et al. [21] in a Cochrane meta-analysis evaluating 7586 patients included in six different studies (ELIOT, GEC-ESTRO, TARGID, LIVI 2015, POLGAR 2007, RAPID), acute dermal toxicity, including radiodermatitis, was less frequent after PBI/APBI (partial breast irradiation/accelerated partial breast irradiation) (OR 0.04, 95% CI: 0.02 to 0.09; *p* < 0.00001). However, they observed an increased rate of late dermal toxicity, especially telangiectasia (OR 26.56, 95% CI: 3.59 to 196.51; *p* = 0.001) and skin fibrosis (OR 6.58, 95% CI: 3.08 to 14.06; *p* < 0.00001).

Due to the sometimes discordant results between studies, Hickey et al. were very cautious in their conclusions concerning the tolerability of this type of radiation therapy, especially as the results of their meta-analysis suggest that local recurrences may be more frequent after PBI/APBI than after whole breast irradiation, although no significant difference was observed [HR 1.48, 95% CI: (0.95 to 2.29)].

PBI/APBI therefore appears to be less effective in terms of local control with a higher rate of late toxicity, but is associated with better tolerability during treatment. However, in view of the discordant results published in the literature, no consensus has yet been reached on this subject.

Intraoperative radiation therapy (IORT) has also been developed to allow irradiation during breast surgery. However, the logistics and infrastructures required for intraoperative radiation therapy have limited generalization of this strategy despite the good results reported by various studies. For example, Key et al. [26] evaluated the dermal toxicity and long-term cosmetic results of intraoperative radiation therapy with a mean follow-up of 38.9 months. They reported 2.4% of grade 2 or higher fibrosis among the 41 patients evaluated. A significantly higher rate of fibrosis was also observed when IORT was associated with WBRT (hypofractionated or normofractionated) with 43.3% of grade 2 or higher fibrosis among the 30 patients evaluated (HR: 0.034, *p* < 0.001). These results were confirmed by the phase 3 TARGIT A study conducted by Sperk et al. [27] in 305 patients with a follow-up of 6 years, in which the rate of grade 2 or higher skin fibrosis was not statistically significant between the IORT and WBRT arms (*p* = 0.984). However, subgroup analyses found an increased rate of grade 2–3 skin fibrosis in the IORT + WBRT arm (37.5% vs. 5.9% for IORT (20 Gy) and 18.4% for WBRT (46–50 Gy) (*p* = 0.008).

IORT therefore appears to provide good results in terms of both local control and late toxicity, but the IORT + WBRT combination significantly increases the risk of fibrosis, and the technical and human resources required for implementation of this strategy constitute a real obstacle to more generalized use.

Apart from modifying the radiation technique, changing the patient’s positioning can also have an impact on toxicity. Some teams have studied radiation therapy delivered in the lateral decubitus position [2]. Bronsart et al. [29] evaluated the efficacy and safety of breast radiation therapy in the isocentric lateral decubitus position in 832 patients with a median follow-up of 6.4 years. Acute and late toxicity rates were very low: 2.8% of grade 3 radiodermatitis and 4.3% of grade 2/3 fibrosis during the study.

In addition to modifying the dose distribution, especially by reducing the dose to organs at risk, ensuring that almost no dose is delivered to the lungs and the heart, radiation therapy in the isocentric lateral decubitus position induces little few acute or late toxicity. However, the patient eligibility criteria for this technique (large, mobile breasts, etc.), the need to have trained radiotherapy technologists to maintain correct repositioning, as well as a longer overall treatment time, make it difficult to generalize this technique.

### 3.3. Roles of Fractionation and Boost

Apart from modifying radiation techniques and developing new techniques, many authors have also studied modified fractionation in the management of breast radiation therapy. The conventional protocol comprising 50 Gy in 25 fractions has been progressively replaced by hypofractionated protocols [30,31], such as 40.05 Gy in 15 fractions or, more recently, 26 Gy in 5 fractions [32]. Another issue concerns the role of a boost [33,34], which can be either integrated or sequential [14]. In addition to the safety and efficacy of these new fractionation modes, some authors have therefore also evaluated the toxicities of these new protocols (Table 2). Offersen et al. [35] compared the toxicity of normofractionated radiation therapy with 50 Gy in 25 fractions vs. hypofractionated radiation therapy with 40 Gy in 15 fractions in 1854 patients with a median follow-up of 9 years after radiation therapy of local or in situ breast cancer without lymph node invasion. The radiation-induced fibrosis rate was higher in the normofractionated arm vs. the hypofractionated arm (OR 0.80, *p* < 0.029) at 9 years of follow-up, but was not significantly different at 3 years and 5 years of follow-up. Other studies confirmed these results by showing a trend towards decreased acute and late toxicity in patients treated by hypofractionated protocols [36,37].

Delivery of a boost dose to the lumpectomy bed also raises the possibility of increased toxicity due to the larger treatment volume. Bartelink et al. [34] studied the incidence of fibrosis in patients with stage 1–2 breast cancer receiving a 16 Gy boost in 8 fractions during 50 Gy breast radiation therapy in 25 fractions. In a population of 2657 patients with a median follow-up of 20 years, the cumulative incidence of moderate to severe fibrosis was higher in patients who received a boost dose [5.2% (99% CI: 3.9–6.4) in the boost group vs. 1.8% (99% CI: 1.1–2.5) in the no boost group (*p* < 0.0001)].

Other authors have confirmed these findings and have also shown that a boost increases the risk of radiodermatitis during treatment [33,38,39].

**Table 2:** Acute and late toxicities depending on the number of fractions.

Author	No. of Patients	Cancer Location	Radiation Dose (Gy) and No. of Fractions (*)	Boost Dose (Gy)	Radiodermatitis ≥ Grade 2	Fibrosis ≥ Grade 2	Cosmetic Satisfaction ≥ Good
Offersen et al. [35]	1854	breast cancer	50 (25) vs. 40 (15)	NA	NA	13% vs. 11% (*p* < 0.029)	90% vs. 91% (*p* < 0.48)
Wang et al. [37]	729	breast cancer	50 (25) vs. 40 (15)	10 vs. 8.7	7.4% vs. 3% (*p* < 0.019)	8.2% vs. 7.9% (*p* < 0.69)	88.7% vs. 89% (*p* < 0.39)
Wang et al. [36]	810	breast cancer	50 (25) vs. 40 (15)	NA	8% vs. 3% (*p* < 0.0001)	0% vs. 1% (*p* < 0.67)	NA
Bartelink et al. [34]	5318	breast cancer	50 (25)	16 vs. 0	NA	4.4% vs. 1.6% (*p* < 0.0001)	NA
Bartelink et al. [33]	2657	breast cancer	50 (25)	16 vs. 0	NA	5.2% vs. 1.8% (*p* < 0.0001)	NA
Palumbo et al. [39]	218	breast cancer	42.4 (16)	10.6–13.25	18.8%	2.3%	NA
Pealinck et al. [40]	167	breast cancer	40.05 (15)	10–14.88	45% vs. 27% (*p* = 0.037)	NA	NA
Brunt et al. [41]	189	breast cancer	40 (15) vs. 27 (5) vs. 26 (5)	NA	51% vs. 29% vs. 36%	NA	NA
Murray et al. [32]	4096	breast cancer	40 (15) vs. 27 (5) vs. 26 (5)	10–16	NA	4% vs. 7.4% vs. 5.6%	70.3% vs. 69.6% vs. 73.3%

Another unresolved issue concerns the impact of the boost sequence, integrated or sequential, on toxicity. Paelinck et al. [40] compared acute toxicity between 40 Gy hypofractionated radiation therapy in 15 fractions associated with a 10 Gy sequential boost in four fractions or a 14.88 Gy boost in six fractions depending on the surgical margins and an integrated boost of 46.8 or 49.95 Gy also depending on surgical margins. This study demonstrated a statistically significant difference among the 167 patients evaluated between the sequential boost arm and the integrated boost arm with a higher rate of grade 2–3 radiodermatitis in patients treated by sequential boost (38/83 vs. 24/83 patients, *p* = 0.037). However, there was no significant difference between the two arms in terms of moist desquamation (*p* = 0.071).

Hypofractionated regimens are widely used in Scandinavian countries and England and are the main drivers in the search for new protocols designed to reduce the overall treatment time. Several studies have evaluated the efficacy, safety, and tolerability of five-fraction breast radiation therapy [32,41]. In the FAST Forward trials, Brunt et al. [41] evaluated the acute toxicity observed with various hypofractionated regimens, 40 Gy in 15 fractions, 27 Gy in 5 fractions, or 26 Gy in 5 fractions. A total of 189 patients, randomised 1:1:1 between the three protocols, were evaluated during treatment and up to 4 weeks after the end of radiation therapy. No major difference was observed in terms of tolerability or the incidence of radiodermatitis between the three groups. Grade 3 toxicity, evaluated according to RTOG criteria, was reported in 13.6% of patients in the 40 Gy/15F group, 9.8% in the 27 Gy/5F group, and 5.8% in the 26 Gy/5F group. However, according to CTCAE criteria, the proportions of evaluable patients with grade 3 toxicity were: 0% in the 40 Gy/15F group; 2.4% in the 27 Gy/5F group, and 0% in the 26 Gy/5F group. Acute toxicity was therefore slightly more frequent in the 27 Gy/5 fractions group.

Modified fractionation can therefore have an impact on acute and late toxicity, as the literature tends to argue in favour of hypofractionation with equivalent efficacy and safety to the normofractionated regimen, but with lower rates of radiodermatitis and fibrosis. The adddition of a boost, particularly a sequential boost, increases the risk of fibrosis and radiodermatitis during treatment. New, even more intensely hypofractionated regimens, are also emerging, including five-fraction regimens that have been reported to be associated with low acute toxicity in recent studies.

### 3.4. Predictive Factors (Genetic, Environmental, Epigenetic)

Potential genetic, environmental, and epigenetic predictive factors for radiation-induced toxicity have been extensively evaluated [42,43,44,45,46,47]. Kraus-Tiefenbacher et al. [43] conducted a retrospective study to identify factors influencing acute skin toxicity during 3D conformal radiation therapy with a protocol comprising 50 Gy in 25 fractions associated with a 16 Gy boost in 8 fractions in 211 patients. After evaluating a large number of different factors, smoking during treatment (*p* = 0.034), large breast volume (*p* = 0.003), and absence of allergy (*p* = 0.002) were associated with an increased risk of acute skin toxicity in multivariate analyses. These results were confirmed, in particular, by Sharp et al. [46], who showed that smoking constitutes an independent risk factor for severe radiodermatitis during adjuvant radiation therapy for breast cancer.

Some authors have also studied potential risk factors for the development of long-term fibrosis [10,48]. Collette et al. [49] evaluated 3624 patients irradiated for breast cancer with 50 Gy ± 16 Gy with a median follow-up of 10.7 years. They demonstrated a statistically significant association between the risk of fibrosis and the maximum whole breast irradiation [HR 1.24 (1.14; 1.35)] and concomitant chemotherapy [HR 2.52 (1.38; 4.62)]. The risk of fibrosis in the boost arm was further increased when the patient presented postoperative oedema or haematoma (*p* < 0.01).

De Santis et al. [42] evaluated the risk factors for radiodermatitis and fibrosis in 337 patients with a 5-year follow-up treated according to a hypofractionated regimen of 42.4 Gy ± 2.65 Gy. In contrast with previous studies, De Santis did not identify any predictive factors, such as breast volume, total dose received by the patient, or even concomitant chemotherapy, but showed that a boost dose increased the risk of radiodermatitis (OR: 1.9, *p* < 0.02) without increasing the risk of fibrosis (OR: 1.5, *p* < 0.24).

Other studies have evaluated the role of genetic factors in the development of radiation-induced toxicities [50,51,52,53]. Based on a review of the literature, Lazzari et al. [53] evaluated the risks of increased acute or late toxicity depending on the presence of BRCA, ATM, and P53 mutations. No increased radiosensitivity or acute or late toxicity in patients with BRCA mutation has been reported in the literature. These data were confirmed by Shanley et al. [51], in a retrospective study evaluating 110 patients with BRCA1/2 mutation, which did not find any significant association with radiodermatitis or fibrosis. The presence of a P53 mutation is considered to be deleterious in terms of radiosensitivity, but its impact on acute or late clinical toxicity is difficult to assess due to limited data on this subject, mainly consisting of in vitro data. The impact of ATM mutation on radiosensitivity and toxicity has not been clearly established, as it is highly dependent on the type of mutation and the various associated mutations.

Many studies have tried to identify individual, genetic, and environmental predictive factors. However, due to the discordant data of the literature, no predictors of toxicity have been clearly identified, but there is a trend towards a consensus concerning smoking and breast volume as risk factors for toxicity. No genetic factor is currently considered to be a predictor of toxicity, essentially due to the limited data available.

### 3.5. Treatment and Adjuvant Techniques (Creams, Dressings)

Many authors have assessed the value of adjuvant treatments, such as topical creams, lotions, gels, or dressings, to prevent the risk of radiodermatitis during breast radiation therapy. Chargari et al. [54] reviewed all topical treatments used for the prevention or management of radiodermatitis and showed that some of these treatments can have harmful effects, such as *Aloe vera*, which increases skin toxicity. Furthermore, no topical treatment (topical corticosteroids, *Calendula officinalis*, etc.) has been shown to be superior to trolamine. However, the small sample sizes of these studies, the poor distinction between preventive and curative treatment, and inter- or even intra-observer variability in the evaluation of radiodermatitis make it difficult to interpret these data, although encouraging results have been reported for some treatments, such as hyaluronic acid.

In a study comprising 200 patients, Kirova et al. [55] did not observe any significant difference in terms of efficacy between hyaluronic acid and simple emollient for the management of radiodermatitis.

Other authors have assessed the use of dressings in the management and prevention of radiodermatitis [56,57]. Bazire et al. [58] studied the efficacy of Hydrosorb^®^ (hydrocolloid dressing) in the management of grade 1 and 2 radiodermatitis. Hydrosorb^®^ did not improve the management of radiodermatitis compared to a water-based spray.

A large number of studies have evaluated adjuvant treatments in the prevention and management of radiodermatitis. However, the small sample sizes and inter- and intra-observer variability despite implementation of CTCAE v4 criteria make it difficult to interpret the results of these studies. No local treatment has been shown to be superior at the present time.

### 3.6. New Methods of Evaluation of Radiodermatitis (RILA, Ultrasound, Spectrometry)

One of the current challenges in the management of radiation-induced toxicities is the prevention and the precise and objective quantification of acute and late adverse reactions.

Possible predictive tests of toxicity, particularly fibrosis, have been studied for many years, including the comet assay [59], clonogenicity test [60], detection of the apoptosis rate, etc., but none of these tests has shown to be sufficiently robust for use in routine clinical practice.

However, the radiation-induced lymphocyte apoptosis test (RILA) [61], which assesses the individual risk of grade ≥ 2 fibrosis, has provided promising results. By determining the CD8 T lymphocyte count after delivering 8 Gy of irradiation to a blood sample, the RILA test has a negative predictive value of 91% for the risk of grade ≥ 2 fibrosis for a CD8 count ≥ 20% and a positive predictive value of 22% for a CD8 count < 12%. At 3 years, a RILA test < 12% is predictive of a cumulative incidence of grade ≥ 2 fibrosis of 19% (95% CI: 14–26%), a RILA test between 12–20% is predictive of a cumulative incidence of grade ≥ 2 fibrosis of 9% (95% CI: 5–15%), and a RILA test ≥ 20% is predictive of a cumulative incidence of grade ≥ 2 fibrosis of 7.5% (95% CI: 4–13%).

The major advantage of the RILA test is its high negative predictive value. The choice of radiation technique and delivery of a boost dose to a patient can therefore be guided by the individual risk of grade ≥ 2 fibrosis.

Bourgier et al. [62] also assessed 36-month breast fibrosis-free survival (BFFS) in 456 patients treatment by hormone therapy based on their RILA status and showed that patients with RILA < 12% and hormone therapy (tamoxifen or aromatase inhibitor) had a BFFS of 75% vs. 100% in the RILA > 12% group without hormone therapy (*p* = 0.004, hazard ratio 5.84 [95% CI: 1.8–19.1]). The 36-month BFFS was comparable in patients with a RILA test > 12% with hormone therapy and in patients with a RILA test < 12% without hormone therapy (89.8% and 93.5%, respectively; *p* = 0.39, hazard ratio 1.7 [95% CI: 0.51–5.65]).

Several studies have assessed the methods that allow more accurate objective evaluation of radiation-induced skin toxicity [61,62,63]. Yoshida et al. [62] investigated the use of spectrophotometry and ultrasound to assess skin fibrosis. Ultrasound measurement of skin thickness and Pearson coefficient allowed quantitative assessment of fibrosis compared to the contralateral breast. In a group of 18 women treated for breast cancer, 50 Gy ± 10–16 Gy with a median follow-up of 22 months after radiation therapy, average thickness of the treated breast skin was 2.61 mm, while that of the untreated breast skin was 2.05 mm. A mean increase of 27.3% in skin thickness was observed (*p* < 0.001). Skin thickness increased by 38.4% for patients with RTOG grade 0, 23.8% for grade 1 patients, and 31.1% for patients with grade 2 toxicity. In contrast, the average Pearson coefficient for the treated breast was 0.28 vs. 0.41 for the untreated breast, corresponding to a mean decrease of 34.1% (*p* < 0.001). The Pearson coefficient decreased by 18.4% for RTOG grade 0, 35.0% for grade 1, and 42.6% for grade 2 toxicity. However, spectrophotometry parameters, melanin, and erythema were not correlated with the development of fibrosis.

These results were confirmed in more detail by Landoni et al. [63], who assessed ultrasound to evaluate skin fibrosis after radiation therapy in 89 women with a median follow-up of 20.5 months after hypofractionated breast radiation therapy at 34 Gy ± 8 Gy. The mean increase in skin thickness relative to the contralateral breast was 0.52 ± 0.67 mm and 0.62 ± 0.74 mm for the treated breast and the boost region, respectively. A significant correlation was found between the increase in skin thickness in the irradiated breast and in the boost region with fibrosis (Grade ≥ 1). The authors suggest that late skin reactions can be reliably assessed by ultrasound, which is also able to discriminate regions irradiated at different doses, including the boost region, in which the risk of fibrosis is increased.

Current research is therefore tending towards implementation of predictive tests of fibrosis such as the RILA test, which has a good negative predictive value for radiation-induced fibrosis, but routine use of this test remains controversial and difficult to implement. New ultrasound-based methods are also being developed to allow objective and precise assessment of skin fibrosis.

## 4. Discussion

Technological progress in the field of radiation therapy has improved treatment tolerability by significantly reducing side effects. The development of IMRT [1] has significantly reduced acute toxicity and improved tolerability during treatment. The introduction of other techniques, such as the lateral decubitus position [2] or deep inspiration breath hold [3,4], has also allowed a reduction of the dose delivered to organs at risk, thereby reducing potential toxicities. Modified fractionation [32,37,41], especially for the treatment of breast cancer, has reduced treatment time, as well as adverse effects, especially the risk of fibrosis. However, the addition of a boost dose increases the risk of acute and late toxicity, especially in the case of sequential boost.

The management of radiodermatitis is not standardized at the present time and no topical treatment has been shown to be superior, either curatively or preventively [54]. Identification of risk factors for poor tolerability of radiation therapy would allow treatment adaptation in patients at risk. At the present time, no genetic factors, such as BRCA, ATM, or P53, have been demonstrated to be associated with an increased risk of acute or late toxicity [50,52,53]. Smoking, large breast volume, and delivery of a boost appear to be emerging as risk factors for skin toxicity, but the literature on this subject remains discordant. Many tests have been evaluated to classify the risk of fibrosis in a given patient, but remain difficult to apply in routine clinical practice. To date, only the RILA test [59] appears to be promising, but still needs to be validated before becoming part of routine practice. Ultrasound [62] appears to be a reliable and reproducible technique to characterize and evaluate radiation-induced fibrosis by measuring skin thickness and the Pearson coefficient.

New technique are decreasing the skin reactions. For example, Donovan et al. [64], showed that the majority of intensity modulation techniques improved dose distribution in breast volume by 5.6% to 11.1% (mainly in women with breasts of 500 cm^3^ or larger).

Many challenges have yet to be resolved in order to improve the tolerability of radiation therapy, either concerning the radiation technique, with the growing use of stereotaxic techniques or particle therapy, or by improvement of curative or preventive treatments of fibrosis. The identification of genetic and environmental factors would also allow tailored treatment guided by predictive tests prior to radiation therapy. The dermal toxicity of radiation therapy has now been extensively described and today’s challenge is to ensure better prevention, evaluation, and reduction of the risk of radiodermatitis.

At the other hand, tt is important to note that the evaluation of skin toxicity remains subjective and may be discordant between the evaluation of the practitioner and that of the patient [65], like was the case in the recall of the Cambridge trial [66].

## 5. Conclusions

The evolution of radiotherapy in recent years responds to the fundamental need to improve patient management. In parallel with the introduction of new, more effective treatment modalities through a combination of techniques or treatments, tolerance and the occurrence of side effects constitute, today, preliminary data regarding the implementation of a new treatment protocol. Today, the evaluation of the efficacy and safety of radiotherapy is inseparable. Faced with the multiplicity of new treatment modalities (SBRT, IMRT, Hadrontherapy, etc.), new fractionation patterns (normofractionated, hypofractionated, ultra hypofractionated, etc.) emerge.

The evaluation of toxicities becomes essential especially as they can occur several months or years after irradiation.

Today, many players are looking for a way to prevent, treat, and quantify the occurrence of these toxicities. However, to date, no method, test, or treatment is the subject of consensus and use in current practice. Much work is underway, particularly in the field of genetics and epigenetics, in order to understand and act on radiation-induced toxicity.
